# Barriers and facilitators to COVID-19 testing and vaccination: a qualitative focus group study among Rhode Island’s Latine/Hispanic community

**DOI:** 10.3389/frhs.2025.1473375

**Published:** 2025-07-29

**Authors:** D. Grace Smith, Yovanska Duarte-Velez, Elizabeth S. Chen, Mario Bueno, A. Rani Elwy, Indra Neil Sarkar

**Affiliations:** ^1^ Advance Clinical and Translational Research, Division of Biology and Medicine, Brown University, Providence, RI, United States; ^2^ Department of Psychiatry and Human Behavior, Bradley Hospital, Riverside, RI, United States; ^3^ Department of Psychiatry and Human Behavior, Brown University, Providence, RI, United States; ^4^ Center for Biomedical Informatics, Brown University, Providence, RI, United States; ^5^ Progreso Latino, Central Falls, RI, United States; ^6^ Rhode Island Quality Institute, Providence, RI, United States

**Keywords:** COVID-19, Latine/Hispanic, implementation science, health disparities, health behaviors, qualitative methods

## Abstract

**Introduction:**

Due to a combination of cultural, situational, social, and environmental factors, members of the Latine/Hispanic community experienced higher contagion and poorer outcomes amid the COVID-19 pandemic, and lower rates of testing and vaccination. Our aims were to use the frameworks of implementation science to identify barriers and facilitators impacting equitable access to COVID-19 testing and vaccination programs among Rhode Island's (RI's) Latine/Hispanic community.

**Methods:**

Between February and June 2021, we implemented a community-centered approach, empowering Promatoras, trusted community health leaders, to conduct eight focus groups among RI's Latine/Hispanic community (*n* = 55). To gain the perspectives of health delivery experts, we conducted six one-on-one interviews with healthcare professionals serving this community. Recordings were translated into English as applicable, transcribed, and analyzed using directed content analysis and thematic analysis, guided by theories of implementation science.

**Results:**

Latine/Hispanic community members made decisions about participation in testing and vaccination programs by considering factors primarily related to their communal, religious, interpersonal, and emotional contexts. The amount and sources of information they received, perceived accessibility of interventions, and their perceived agency (i.e., freedom to decide how/when/where to follow interventions) also shaped decisions. Many barriers identified by clinicians (e.g., structural determinants to access) were not discussed by Latine/Hispanic community members.

**Discussion:**

Facilitators and barriers to test/vaccine implementation were shaped by local communal and individual factors, generally supporting previous arguments on implementation among Latine/Hispanic communities, and revealing the importance of context-specific examinations. In public health pandemic preparedness work, we encourage community-based participatory approaches to identify priorities/barriers and involvement of community leaders to build trust, frame messaging, and disseminate information.

## 1 Introduction

Since being declared a global pandemic in March 2020 (1), COVID-19 has caused the deaths of over 1.2 million U.S. citizens (2). Following larger healthcare trends in the U.S., race/ethnicity was among the strongest predictors of poor COVID-19 outcomes, evidenced by increased rates of infection, hospitalization, and mortality among Black, Indigenous, and People of Color (BIPOC) communities and those of Latine/Hispanic ethnicity (1–5). Recognizing these inequities, national research and health leaders have called for exploration of disparities relating to COVID-19 prevalence, outcomes, and care access among underserved populations, defined by the NIH as those who, because of biological, environmental, or systemic factors, were at heightened risk for COVID-19 complications or had limited access to necessary precautions (6).

Rhode Island (RI) is the second most densely populated and the most geographically compact state in the U.S., and it has a Latine/Hispanic population (16%) comparable to the national average (18.7%) (7, 8). It therefore provides a unique opportunity to examine factors contributing to COVID-19 disparities among Latine/Hispanic communities (7, 8). Reflecting national trends in racial and ethnic health disparities, inequities in COVID-19 outcomes were evident in RI as early as April 2020, when the Latine/Hispanic population accounted for 45% of the state's positive COVID-19 tests (8) and experienced higher rates of ICU admission and intubation (3). Latine/Hispanic diagnosis rates continued to be disproportionately higher through the completion of statewide data collection in March 2021 (2, 9).

The most effective public health interventions for preventing COVID-19 included social distancing and quarantining, mask-wearing, contact tracing, testing, and, upon development, vaccination (10, 11). Given the importance of these strategies, particularly among disproportionately-affected populations, previous research has focused on vaccine-related hesitancy and has identified lack of information, fear and mistrust, structural barriers, and doubts of efficacy as key barriers among (typically combined) Latine/Hispanic and African American populations (12–16). For example, one recent paper employing community-based research methods to examine the experiences of Latine/Hispanic, African American, and Native American individuals has identified the central role of communication techniques in addressing fear, mistrust, and context-specific barriers (16).

In the research that has been published specifically on the experience of Latine/Hispanic communities during the COVID-19 pandemic, many groups of scholars have focused on emotional well-being (17), or on the relationship between the pandemic and co-occurring experiences, such as cancer or pregnancy (18, 19). Likewise, few previous studies on COVID-19 outcome disparities and vaccine hesitancy have prioritized Latine/Hispanic community-member perceptions, often focusing on larger political, health (e.g., pregnancy), or psychological factors (13–15, 20, 21). Further, few studies have explored barriers and facilitators to COVID-19 prevention protocols more broadly (beyond vaccines; including distancing, tests, and masks), information crucial to crisis preparedness (13–15, 20, 21).

In prioritizing community-member voices, our main aims were to: (1) understand the experiences of information access, supply access, and emotional support among RI's Latine/Hispanic community throughout the COVID-19 pandemic and (2) identify barriers and facilitators to accessing and participating in related interventions. We supplemented this with the perspectives of clinical experts serving the Latine/Hispanic community to identify gaps in understanding and infrastructural factors. Considering together the emotional and access experiences of members of the Latine/Hispanic community, with the perspectives of local experts who worked directly with this community, we hope to identify exact informational and intervention junctures where misinformation and mistrust emerged in order that communication and action protocol for future pandemics might prevent these barriers to well-being. Our findings will facilitate called-for improvements in health crisis preparedness infrastructure, policy developments, and communication campaigns to support more equitable outcomes by both empowering agency and facilitating trust in the future (22).

## 2 Methods

### 2.1 Overview

The National Institute on Health's RADx-UP initiative includes research projects across the U.S. studying COVID-19 testing, vaccination, and outcomes among underserved populations (6). This RADx-UP study was part of a larger statewide tracking, exploratory, and implementation effort aiming to (1) leverage health information infrastructure to study COVID-19 test/vaccine access and uptake in RI, (2) identify barriers to uptake, and (3) identify opportunities to address these barriers via a community-based approach (47). This manuscript reports on the second of these aims. The Brown University Institutional Review Board approved all research procedures.

### 2.2 Procedure

Using a community-centered model, researchers worked closely with Promotoras at Progreso Latino, RI's largest Latine/Hispanic-centered non-profit organization, who guided the development of focus group scripts and, using flyer-distribution, direct communication at events, and word-of mouth, recruited Latine/Hispanic community members. Promotoras (commonly referred to in English as Community Health Workers) are trusted community leaders who promote health, social justice, and equitable care in Spanish-speaking communities (23–25). Previous studies have shown Promotora-empowerment to be effective toward improving a range of health outcomes in Latine/Hispanic communities (23, 26, 27). Promotoras received comprehensive training, including formal CITI Program certification in human subjects research. Additionally, weekly meetings reinforced methodological rigor through debriefing focus groups, refining questions, and emphasizing ethical data collection practices. After being trained in facilitation, Promotoras conducted virtual focus groups (1–1.5 h long) between February and June 2021, to gather community-member perspectives on the COVID-19 pandemic generally and experiences of testing and vaccination specifically; see Table 1. Participants were deemed eligible based on RI residency, age (18+), and Latine/Hispanic identity. They provided informed consent through registration via the video conferencing platform and verbal agreement. Participants received compensation for their time, including financial compensation and the opportunity to ask questions of local health representatives. To minimize bias, focus groups were conducted in Spanish between community members and Promotoras. Research team members observed focus groups without participating and without visual presence.

**Table 1:** Community member focus group key questions.

Number	Focus group question
1	Where do you get your information about COVID-19?
2	What are your feelings about getting tested for COVID-19?
3	What has been your experience with the COVID-19 test? If you have not done it, how would you get tested?
4	When you got the COVID-19 test done, what do you think if the best way to obtain the results?
5	What does a positive or negative COVID-19 test mean to you? *Probes: Did your behavior change after receiving a negative or positive result? What have you seen in your community about how other people behave after a positive COVID-19 result?*
6	What do you think or feel about the COVID-19 vaccines?

To supplement community-member responses, local health professionals who worked with the Latine/Hispanic community participated in virtual one-on-one semi-structured interviews, lasting 20–30 min, between March and May of 2021. Interviews were conducted by an experienced community practice facilitator and interviewees were all clinicians at local Federally Qualified Health Centers or Free Clinics serving high proportions (>50%) of Latine/Hispanic patients. Eligibility requirements included being clinically active and regular engagement with Latine/Hispanic patients. Interviewees provided verbal consent and were not compensated. The purpose of interviews was to gain clinician perspectives on key issues and experiences faced delivering tests/vaccines to the Latine/Hispanic community; see Table 2.

**Table 2:** Clinician interview key questions.

Number	Interview question
1	How important is it for your patient panel to get tested for SARS-CoV-2 or COVID-19?
2	What are the major challenges in ordering COVID-19 tests for your patients? *Probes: What were major challenges at the beginning of the pandemic? What are major challenges now?*
3	How do you get your results for patients who have been tested for COVID-19? *Probes: Which lab system are you connected to for your results? How does your electronic health record system work?*
4	What clinical guidance do you offer patients based on the test results?
5	Do you think the availability of a COVID-19 vaccine is or will affect patients getting tested for COVID-19?

The leadership group and Promatoras, who conducted the focus groups and actively guided analysis discussions, determined data saturation collaboratively, rather than through a formal matrix. By later weeks, our weekly discussions, consistently revealed recurring themes across the focus groups, with little new information, indicating saturation in our understanding of participants’ experiences. Throughout the study, we addressed the trustworthiness criteria of credibility, dependability, confirmability, and transferability. To enhance credibility, we prioritized prolonged engagement and iterative discussions between researchers, Promatoras, and focus group transcripts, which ensured findings accurately reflected participant experiences. We defined dependability as consistent application of the research protocol and maintenance of data quality, and supported this through consistent weekly meetings, structured debriefings, and supervision of the Promotoras. We established confirmability hand-in-hand with saturation, by collaboratively identifying the consistency of themes across the focus groups, and we supported transferability by providing a rich context through detailed discussions of our study processes, enabling readers to assess the applicability of our findings to similar settings.

### 2.3 Data analysis

Focus group and interview data, recorded through the video conferencing platform, were transcribed and translated using a third-party secure professional translation service and analyzed in two phases. First, to identify barriers and facilitators to intervention uptake, we conducted a directed content analysis, with existing theory guiding (deductive) coding, and uncoded content prompting the development of inductive codes (28). The original Consolidated Framework for Implementation Research (CFIR) (24) domains and corresponding subconstructs informed our coding framework. The CFIR's five domains are: (1) intervention characteristics, (2) outer policy context, (3) inner context, (4) characteristics of individuals targeted in implementation, and (5) implementation process (29). Analysts individually coded each transcript using this framework and then, to maintain consistency and rigor, groups of 2–3 analysts cross-coded the transcripts. Because our priority in this work was to maintain a community-centered focus of analysis and empower the perspectives of the members of the Latine/Hispanic community and those who serve them, we held the CFIR categories loosely. In conversations among analysts and prioritizing the data, we redefined each of the categories so that they aligned with the main ideas articulated in CFIR texts, but were flexible and changed meaning to best accommodate the realities of the participants. Where there was tension between category definition and data, the data took priority, and we adjusted the definition, documenting changes in audit memos and a shared codebook (Table 3). The first author led the consensus process by which final coding decisions were made and entered coded data into the qualitative data management software NVivo (30).

**Table 3:** Consolidated framework for implementation research (CFIR) selected codes and inductive codes.

Code ID	CFIR/inductive domains and code name	Code definition
1	Intervention characteristics	
1B	Evidence strength and quality	Stakeholders’ perceptions of the quality and validity of evidence supporting the belief that the intervention will have desired outcomes.
1C	Relative advantage	Stakeholders’ perception of the advantage of implementing the intervention vs. an alternative solution.
1F	Complexity	Perceived difficulty of implementation, reflected by duration, scope, radicalness, disruptiveness, centrality, and intricacy and number of steps required to implement.
1G	Design quality and packaging	Perceived excellence in how the intervention is bundled, presented, and assembled.
1H	Cost	Costs of the intervention and costs associated with implementing that intervention including investment, supply, and opportunity costs. *In this context, cost was understood as non/barrier to intervention for participants.*
2	Outer setting	
2A	Patient needs and resources	The extent to which patient needs, as well as barriers and facilitators to meet those needs are accurately known and prioritized by the organization.
2D	External policy and incentives	A broad construct that includes external strategies to spread interventions including policy and regulations (governmental or other central entity), external mandates, recommendations and guidelines, pay-for-performance, collaboratives, and public or benchmark reporting.
4	Characteristics of individuals	
4A	Knowledge and beliefs about the intervention	Individuals’ attitudes toward and value placed on the intervention as well as familiarity with facts, truths, and principles related to the intervention.
4B	Self-efficacy	Individual belief in their own capabilities to execute courses of action to achieve implementation goals.
4C	Individual stage of change	Characterization of the phase an individual is in as he or she progresses toward skilled, enthusiastic, and sustained use of the intervention.
4E	Other personal attributes	A broad construct to include other personal traits such as tolerance of ambiguity, intellectual ability, motivation, values, competence, capacity, and learning style. *In this context, used to refer to expressions of high emotional valence.*
6	Inductive codes: social/societal influence	
6A	Trust	Level or aspects of trust in the implementation, intervention, process of delivery, related leaders/perspectives, etc.
6B	Misinformation	References to conspiracies, rumors, ’stereotypes,’ biased news stories, etc.
6C	Sources of information	References to the sources from which participants receive information about the intervention, about implementation, and about the pandemic.
6D	Equity	References to in/accessibility of intervention, geographic or racial disparities, biases, etc.
6E	Religion	References to faith, God, religious leaders, and religious communities as they relate to attitudes toward intervention and COVID-19.
6F	Interpersonal influence	References to any consideration (including the beliefs, actions, protection, or interactions) of others with regard to decisions about participating in the intervention.

Applicable codes from the Consolidated Framework for Implementation Research (CFIR) Coding Framework (in the domains 1, 2, and 4), with notes on their use with this data. Codes labelled as 6 are inductive codes, identified through the Directed Content Analysis process.

The second phase of analysis was thematic (31), examining the coded data for patterns within and across codes towards the development of overarching themes that describe key implementation trends. With guidance and review from a secondary analyst (fifth author), the primary analyst reviewed, defined, and named the themes, and selected illustrative participant quotations (31).

## 3 Results

Eight focus groups were conducted among groups of 3–12 Latine/Hispanic individuals, with a total of 55 participants, 30 females and 25 males. Six supplemental interviews were conducted among female and male practitioners at local clinics.

In the directed content analysis phase, we identified as applicable to the data 11 codes from CFIR subconstructs in three domains: (1) Intervention Characteristics (test and vaccine), (2) Outer Setting (environmental and policy determinants, RI), and (4) Characteristics of Individuals (Latine/Hispanic community-members). Because of our community-centered methods, we inductively generated six codes related to social influences; see Table 3. In the thematic analysis phase, we generated six themes, which are described below, with exemplar quotes in text. The definitions and relevant codes for all themes and subthemes are in Table 4. To protect participant identity, all quotes are de-identified; community member quotes are marked as [CM] and healthcare workers as [HCW].

**Table 4:** Themes, subthemes, and relevant codes.

Theme	Facilitators	Barriers
(1) COVID-19 is a communal experience, and participants made decisions about testing and vaccination in reference to their position within communities and belief systems.	Protection of others (6F)	Negative or non-positive religious associations (6E)
	Positive religious associations (6E)	
	Pressure from Others (6F)	
(2) Further reflecting valuation of community, individuals are influenced by personal and secondhand experiences/stories of testing and vaccination implementation.	Positive first- or second-hand experiences (1B, 4A, 6C, 6F)	Negative first- or second-hand experiences. (1B, 4A, 6C, 6F)
	Witnessing the stakes of the virus (1C, 6C)	
(3) Views and decisions about testing and vaccination were shaped by emotional perceptions and the larger emotional environment regardless of rational information.	“Trust in science” (1B, 6A)	Fear of risks (real and rumored) (1C, 4A)
	Peer pressure against illness or hesitation (6F)	Mistrust in medicine or government (6A, 6C)
		Environment of fear (1C, 4E)
(4) The source, content, amount, and framing of information influenced decisions to participate	Access to valid, reputable information sources or people (6C, 6F)	Misinformation (6B, 6C)
		Overwhelm of identifying true information (4A, 4B, 4E, 6B, 6C)
		Lack of clear information (4A, 6B, 6C)
		Quickly changing information (2D, 6B, 6C)
(5) COVID-19 was a chaotic and uncontrollable time, and participants used various methods to exert control, including choices both to test/vaccinate and to abstain	Travel and work mandates (2D, 4B)	Mandates evoked mistrust; self-confidence in alternatives (2D, 4B)
(6) Participants had different frames of reference for considering privilege/disparities regarding COVID-19, and did not discuss environmental/situational barriers identified by clinician interviewees.	Privilege relative to home country (4E, 6D, 6F)	Only clinicians saw disparate access to information, care. (6D)

Definitions of each of the six themes with brief descriptions of each of the subthemes, according to their role as barrier or facilitator to intervention uptake. The numbers in parentheses for each subtheme are some of the relevant codes that were combined to define the theme (see Table 3 for meaning).

On the whole, participants expressed a range of views regarding COVID-19 testing and vaccination, from trust and enthusiastic participation to mistrust and refusal to participate. Many variables influenced these decisions, including information sources, perceived ease of access, and personal, religious, and communal factors. Clinician interview data largely supported the views expressed in focus groups. Notable contrasts existed in perceptions of risks, barriers, and knowledge access.

### 3.1 COVID-19 is a communal experience, and participants made decisions about testing and vaccination in reference to their position within communities and belief systems

#### 3.1.1 Facilitators

Many participants viewed intervention uptake as advantageous—and worth discomfort, fear, or other risks—because it helped to protect both themselves and their family/community members, particularly those that, because of age or underlying factors, were at heightened risk of harm. Some participants spoke to the inherently communal nature and high stakes of contagion, encouraging others to participate (i.e., towards “herd immunity”) or saying that they had participated in the intervention in response to others.

“Let us remember that there are elderly people, much older people who do not have the strength or the capacity that we have. Therefore, we have to take the test and keep our distance as soon as [inaudible] we find out we are positive. Why? Because of others.” [CM]

Further, when religious beliefs/leaders encouraged the intervention directly or indirectly (e.g., intervention is from God, or is okay because it does not keep us from God's wishes), participants were more likely to partake in masking, distancing, or vaccination.

“I am 100% positive and I think it was a great opportunity that first God gave us, and then the scientists.” [CM]

#### 3.1.2 Barriers

When participants’ religious beliefs/leaders directly dissuaded them from participation or when participants decided to prioritize faith in God over intervention participation (i.e., faith would keep them healthy), they were less likely to participate in the intervention.

“So many people say, ‘I am not going to get vaccinated, because if I get vaccinated, I am doing something to favor the devil.'” [CM]

### 3.2 Further reflecting valuation of community, individuals are influenced by personal and secondhand experiences/stories of testing and vaccination implementation

#### 3.2.1 Facilitators

Almost all participants described personal/secondhand experiences as crucial to their decision-making regarding testing/vaccination. When participants or their community-members had positive experiences (e.g., test results affirming symptom experiences or vaccine providing immunity), they gave both rational and emotional articulations of trust.

“My mother is 90 years old and a month ago my brother got the coronavirus, but my mother has already been vaccinated and they are both alive.. I believe very much in the vaccine and I recommend people who don't believe in it to get vaccinated.” [CM]

Some participants described participating in the intervention with fear, or despite having heard negative stories from others.

“The expectation of what side effect it is going to have on you. Many people would say to you, ‘It put me in bed for four days.’ Another would tell you, ‘It didn't do anything to me.’ ‘I had a lot of pain in my arm.'” [CM]

After stating these fears, many participants shared that they had had a positive personal experience. This in turn led to future participation. Further, some described being motivated to participate in the intervention upon witnessing the severe illnesses of others, realizing the stakes of implementation through others’ experiences.

#### 3.2.2 Barriers

When participants were exposed to first- or second-hand experiences that were negative (e.g., of pain/discomfort, incorrect test results, or vaccine inefficacy), they were dissuaded from initial or repeated participation in the intervention on account of mistrust or fear.

“I had 14 days in isolation in the attic of the house, with all the symptoms that came with it, very strong, very difficult, but the test came back negative. It is somewhat frustrating and disconcerting… I'm definitely 100% sure that I got it. However, the tests came back negative.” [CM]

### 3.3 Views and decisions about testing and vaccination were shaped by emotional perceptions and the larger emotional environment regardless of rational information

#### 3.3.1 Facilitators

Some participants articulated full trust in “science” or belief that the intervention would result in the desired outcome. These occurred most frequently without any further explanation.

“I have been tested several times, I am like a fanatic, because I look for the safety of not infecting others” [CM]

A few participants also described emotional environments of judgement against those who were ill or not testing/vaccinating, encouraging participation.

“At work there is also that panic, if someone sneezes they already have COVID, I have seen at work how two women grabbed each other by the hair because one of them sneezed.” [CM]

#### 3.3.2 Barriers

One of the most significant barriers to testing/vaccination was fear, with participants arguing that participation was not worth the risks involved. Many mentioned both real (e.g., side effects, discomfort, speedy production) and rumored (e.g., death or sterility) risks, and a few described mistrust in the medical institution/government.

“It is practically an invasion of one's nose to try to prove that one has or does not have it.” [CM]

Clinician interviewees affirmed this trend, mentioning hesitancy as a consistent barrier to intervention.

“I think that we're still fighting the hesitancy, right?” [HCW]

Participants described additional contributing emotional factors including the larger panic surrounding COVID-19.

“We were all, in one way or another, in a panic because it created a panic. The panic was so great that people were killing each other for toilet paper. People were going to the supermarkets and there was fighting over toilet paper, something astonishing, something unbelievable.” [CM]

For some, fear prevented intervention participation.

“The stereotypes and everything that people say, that it is going to turn you into a zombie, that it is going to give you I don't know what, that it is going to give you I don't know how many consequences.” [CM]

For others, the impetus to overcome fear facilitated participation, in that participants, observing the chaos around them or experiencing great fear of infection themselves, saw the intervention as their main source of hope and participated accordingly.

### 3.4 The source, content, amount, and framing of information influenced decisions to participate

#### 3.4.1 Facilitators

When participants had access to valid information about COVID-19 (e.g., contagion rates) and about the function and efficacy of tests/vaccines, most consistently via medical or governmental sources, they were more likely to participate in the intervention.

“I receive the information every day since the pandemic started, wherever we go, there is always information about COVID, through social networks, the same way you find out about it through the news, through television, you find out at every moment.” [CM]

Clinician interviewees affirmed this, saying that community members had access to reputable sources of information.

“I think, in terms of what’s being communicated at the state level as far as what procedures to do is pretty clear, and I think people have a pretty good understanding at this point, but always checking in back with us with anything regarding symptoms or what to expect for their health going forward.” [HCW]

#### 3.4.2 Barriers

The most common source of information, by far, was news or social media. Participants described accessing both misinformation, rumors, or other untrue dissuading information and helpful/truthful information from these sources. Many also described the overwhelm resulting from media excess and the difficulty parsing truth from misinformation, preventing trust and participation.

“Unfortunately people take a lot from what other people say and not from authorized sources. That is why so many conspiracy theories have been created, that this is from the devil, that the vaccine, that this is the new world order.” [CM]

Moreover, many clinician interviewees identified access to detailed and comprehensible information as a key barrier, and a few described efforts to provide information to community-members.

“Even, let’s say, they live with family members, I don't think they really understood what quarantine meant, and that meant being in a separate room from everyone else, making sure you're cleaning after yourself.” [HCW]

Despite these efforts (suggesting possible inefficacy or communication barriers), most focus group participants described feeling unsupported by medical professionals/sources, not having enough information, or lacking access to suitable evidence for the intervention’s efficacy. For example, only one of the 55 participants articulated an understanding of the process by which the vaccine works.

“I took care of myself a little bit, but they never told me what I should do. They did not give me any medication, they did not tell me what I should take, what I should not take. I have been really worried about it and I will not forget the way they acted about it, because if so many people are dying, how is it that they did not pay a little attention.” [CM]

Further, because of the real inefficacy, speed, and risks of interventions, information access was a barrier when these disadvantages were taken as more salient than advantages.

“I'm skeptical about the process of the vaccine, the record time in which the vaccine was made, it definitely makes one worry about, why so fast if the tests that have been done are not that many, and it hasn't lasted long enough to know what the long term effects are.” [CM]

### 3.5 COVID-19 was a chaotic and uncontrollable time, and participants used various methods to exert control, including choices both to test/vaccinate and to abstain

#### 3.5.1 Facilitators

Many participants described participating in the intervention on account of travel-based or medical policies mandating it.

“About the vaccine, actually, they told me to get it. They told me, ‘Get it, because you're going to need it like an ID or something. Then to go out or to go into a mall, etcetera. That’s going to be like your social security, this whole residency thing, something like that, the paper they give you when you get vaccinated.’ What I think about the vaccine is that–I've always been afraid to get the vaccine.” [CM]

#### 3.5.2 Barriers

Even as these policies/mandates facilitated participation, some viewed this as a violation of agency and, for this reason, expressed mistrust of the intervention and medical institutions, preventing further participation.

“The one I was given was Johnson’s, I was not given a choice, I have heard that Moderna’s and the others are better than Johnson’s, why is Johnson’s a single dose?” [CM]

Additionally, even as many participants articulated confidence in their abilities to prevent COVID-19, these actions took varying forms, from intervention participation, to medical practices excluding the interventions (e.g., hand-washing or isolation), to religious action and faith.

“What I did do was to take good care of myself. I took a lot of vitamin C and ate a lot of yellow fruits, which is what prevents diseases. The truth is that I have not taken the test throughout the year. I can't say what COVID is, what the test is, nothing like that. I have had no need for any of it.” [CM]

Participants also exerted control in choosing information sources—including medical, social/media, and religious.

### 3.6 Participants had different frames of reference for considering privilege/disparities regarding COVID-19, and did not discuss environmental/situational barriers identified by clinician interviewees

#### 3.6.1 Facilitators

Participants saw themselves as privileged by free/affordable and straightforward access to the intervention and supportive community-members, particularly compared to the costliness or inaccessibility experienced by residents of their home-countries—for some, this relative privilege encouraged participation in the intervention.

“We have a great opportunity here, because in my country, in Colombia, they are not even doing the tests, and if they do, they are very expensive.” [CM]

“It would be great if everyone could have access to the vaccine, like we have here in the United States and not like in our countries where we see that people are dying more and more every day. It is a shame to see that many people here are refusing to get the vaccine, when in our countries people are begging for the vaccine to arrive” [CM]

#### 3.6.2 Barriers

Clinician interviewees saw the Latine/Hispanic community as experiencing significant disparities in resource access, insurance-related barriers, and lack of information, education, and understanding.

“I will say that as far as patients that do come in, that have difficulties with health insurance, it becomes a little challenging, especially when you try and navigate how can we, A, get them tested, and then B, address their health insurance concerns. That makes things a little complex.” [HCW]

“Our population was hit not only the hardest in Rhode Island but actually in the world. It ranked pretty high in terms of positivity rate. There’s a lot of factors that go into that. I'd say the most basic one is the fact that we're a densely populated area, and we are very underserved where we serve a very underserved population, that’s not a secret.” [HCW]

Clinician interviewees also described the elevated risk of COVID-19 contagion faced by the Latine/Hispanic population due to cohousing norms (increasing risk of contagion and making isolation difficult), financial pressures to continue in-person work, lack of transportation/clinical access.

“I think [testing/vaccination is] incredibly important, especially considering people’s dwellings, the amount of contact that people have with each other. A lot of higher level cultural things like religion and people’s affinity for gathering for spiritual purposes, it’s a really big part of our community and our culture. I think, the more testing for our community, the better.” [HCW]

“No matter what you said, they're like, ‘Well, I've got to put food on the tables, got to go to work.’” [HCW]

Notably, no participants mentioned these barriers.

## 4 Discussion

Our research explored facilitators and barriers to the access and use of COVID-19 mitigation interventions among RI's Latine/Hispanic community, and moves beyond previous studies in two important ways. First, in working toward health equity, we centered Latine/Hispanic community-member perspectives in analytic prioritization, use of qualitative methods, and empowerment of Promotoras for design, recruitment, and moderation. This specificity to one minoritized community [rather than combining multiple BIPOC communities, for example (13–16)], allowed for more clear and specific trends, the leadership of Promotoras fostered participant comfort and honesty, and the analysis extended the possibilities of implementation science frameworks by combining inductive with deductive methods. Second, in seeking a broader understanding of facilitators and barriers to crisis intervention generally, we analyzed discussion of vaccines alongside tests, masking, and distancing. We argue that these decisions at the core of the study make it particularly applicable to the development of equitable health crisis preparedness protocol.

Overall, we found that members of the Latine/Hispanic community made decisions about intervention participation in a deeply contextualized way, shaped by beliefs and duties associated with their larger community and by first- and second-hand experiences/influences. We also found that Latine/Hispanic community members made decisions in reference to their emotional instincts and within fear- and mis/trust-laden environments, that some experienced an excess of (conflicting) information and a dearth of medical information, and that many viewed decisions on test/vaccine participation as an opportunity to exert agency within these environments. Finally, we found that Latine/Hispanic community members’ decisions about test/vaccine uptake were influenced by perceived ease of access, and that they did not identify the structural barriers that clinicians named as significant.

Our findings support studies positing that key factors preventing vaccine uptake among Latine/Hispanic and African American communities include fear and mistrust related to incorrect, inconsistent, and lacking information (13, 16, 32, 33). Some scholars have argued that COVID-19 was intertwined with an “infodemic,” marked by excessive, rapidly changing, and conflicting information released surrounding disease outbreak (34, 35), and others have identified the lack of clear information as a direct cause of isolation and uncertainty among Latine/Hispanic, as well as among other, populations (31). These authors propose access to correct information was a facilitator of vaccine uptake or precautionary behaviors, and lacking or incorrect information were barriers (13, 16, 32, 34); our conclusions generally support this claim. One of these studies has argued for the power of anecdotal, personal, examples as an important evidence base for vaccine trust in the general public (33). Our findings on the significance of positive interpersonal influence as a facilitator to intervention uptake supports these findings in the Latine/Hispanic context. However, in our sample, knowledge about true risks of tests/vaccines, or negative interpersonal influence, was a barrier for some, suggesting that medical information must be communicated intentionally, particularly with communities less prone to trust (13, 14). Future health crisis preparedness plans should take into account the complexities of infodemics, the power of interpersonal influence, and their particularly significant impact on Latine/Hispanic communities.

Our findings also establish a deeper understanding of the contexts in which Latine/Hispanic community members received and interpreted information, which we argue are key to their decision-making. Previous studies acknowledge religious, trusted community spaces/leaders, and the experiences of others as important for communicating information and establishing trust (13, 14, 16, 32). Our findings support these claims, and we add consideration of duties to protect and interpersonal relationships. Relatedly, previous explorations of vaccine hesitancy among BIPOC communities have claimed that much mistrust stems from historical exertions of injustice and violence (13, 14, 16, 23). In combining study of multiple minoritized/BIPOC communities in the U.S., studies have generalized these historically-rooted traumas/mistrusts to the Latine/Hispanic community (16). Our results differ from this assumption: while multiple participants in this study voiced doubts in the intervention, and a few mistrust in “science,” in line with previous studies (16), none discussed these histories. We note this not to minimize attention to these histories or claim they have no impact on the Latine/Hispanic community, but to highlight the importance of implementation strategies shaped according to community-specific barriers. Our conclusions suggest that, in contrast with African American, Native American, and combined/generalized communities previously surveyed (13–16), Latine/Hispanic participants in RI experienced fear primarily associated with community environments and social/media information, establishing the importance strengthening community-specific trust and communication networks.

This insight, among others, illustrates the methodological contributions of the study. Even as we used an implementation science framework to structure analysis of the themes, we centered these community-based methods by changing and adding to the framework. This application of both inductive and deductive methods expands the possibilities in implementation science work, allowing local knowledge to lead insights, and eventually implementation, even beyond that typically allowed by the frameworks. For example, we found factors, including interpersonal influence, religious affiliation, and a sense of trust, which the CFIR does not take into account (29), to be central to understanding Rhode Island's Latine/Hispanic community's experience and perspectives. Future implementation science studies, particularly among underserved or minoritized populations, might benefit from similar combinations of deductive and community-centered, inductive, methods.

Using participatory, community engagement methods may also increase future implementation success. Involving interested parties as key members of the research team from the beginning of a project may lead to their involvement as communication and dissemination partners during implementation phases, creating trust with communities in the process (36). Involving community participation on a research team also allows for the co-production of knowledge and solutions which inform implementation strategies to increase the uptake of evidence-based interventions in community settings (37). Just as we included Promotoras as part of our team, future implementation science efforts also need to include trusted members of the community in the research effort, providing a bridge between the research team and implementation efforts in the community.

However, participatory methods often require an inductive approach, as theory-driven methods (e.g., CFIR) may not be well understood by community members on the team. These inductive and theory-driven approaches may complement each other if they are used in a two-step fashion, as our team did with the current study. We first identified the lived experiences of barriers and facilitators to COVID-19 testing and vaccination from community members and health care workers. Through this, we redefined the specific CFIR categories, and added new context-specific categories, to align with these lived experiences. We applied this new framework deductively. In this way, our project used community-driven knowledge to inform future implementation strategies that come from CFIR, a method which informs the selection of implementation strategies for future implementation efforts (38).

Further, many participants mentioned receiving a COVID-19 test or vaccine on account of mandates for travel, medical visits, or work. While previous studies have noted that requirements increase test/vaccine uptake, and thus are crucial to population safety (10), some members of our sample spoke to the importance of granting agency, particularly toward establishing trust. This extension to previous research provides additional evidence for the importance of inductive, community-based prioritizations beyond the deductive, implementation-science-based methods, as it illustrates the complex relationship between trust, experience, and perceptions of agency, beyond those that the CFIR considers (29). This is especially important among communities with lower trust in medical institutions/interventions, and should be taken into account in framing equity-oriented communication in future health emergencies, as we discuss below.

Our final theme described important discrepancies between the Latine/Hispanic community members and local clinicians, not thoroughly discussed in the literature. Notably, members of the Latine/Hispanic community—in contrast to the clinicians serving them—did not describe lack of access to tests/vaccines and did not comment on social and environmental determinants of COVID-19 outcome. Scholars have established that a key source of emotional burden among Latine/Hispanic individuals during COVID-19 was concern about family members living abroad (7), and others have highlighted the significant stress of immigration status and familial vulnerability as significant beyond pandemic-fears (17). Our arguments expand these claims by proposing that, relative to these concerns, perceptions of privilege (particularly among those farther from borderlands or less directly impacted by immigration threats 17) motivated testing/vaccination. Additionally, participants perceived a lack of communication from medical professions, discordant with clinician attempts to educate. Noting these discrepancies and the prevalence of public health research shaped within positions of power, as opposed to the community-centered perspectives we strive for here, we urge further study into the perceptions of privilege, priorities, and barriers among Latine/Hispanic communities to establish stronger collaboration models in addressing inequalities.

### 4.1 Limitations

In sharing these results, we describe only a portion of RI's Latine/Hispanic community. While we hope that the richness of these findings will be helpful in developing more equitable health crisis preparedness plans more broadly, we do not claim these results to be generalizable (27). Further, our data collection methods brought limitations insofar as, outside of focus groups, but as for their time, participants were given the opportunity to ask questions of state health leaders. This might have created pressure influencing participants’ sharing. To mitigate impact, these leaders, when applicable, left before the focus group began, and Promotoras repeatedly emphasized confidentiality and welcomed all opinions. Open expressions of disagreement or mistrust of interventions suggests that these measures were, at least in part, successful, but we acknowledge that other participants may have not shared as freely as hoped because of research team presence. We also recognize that trends particular to COVID-19 tests or vaccines may have been missed because we analyzed discussion of both interventions together, preventing intervention-specific implementation recommendations.

Furthermore, we did not collect racial information, using only Latine/Hispanic ethnic identity to reflect the population prioritized by RIPIN; this neglects differences among possible variations in racial self-identification. Prioritizing the safety and comfort of participants, we also did not collect citizenship information. This prevented examination of insurance- and other status-related fears and barriers to the intervention, previously identified as key determinants of COVID-19 outcome and care access (7, 13, 39, 40). While we believe this was the correct approach for our study, it limits the scope of our conclusions, as citizenship status may be an unrecognized mediating factor (39, 40).

## 5 Conclusions

Adjusting implementation science methods of analysis to center the voices of RI's Latine/Hispanic community, we have discussed key barriers and facilitators to test/vaccine implementation. Taken together, our findings contribute both to COVID-19-specific considerations, and to the broader implementation of public health interventions in the Latine/Hispanic community, by exploring the wide range of interpersonal, belief-based, and global contexts within which community members make personal health decisions. Even as our Latine/Hispanic specific findings differ in important ways from a published community-based participatory study among minoritized populations, our results emphatically support their conclusion that cultural and environmental contexts are central to understanding medical beliefs and decisions (16). These authors have argued for the implementation of further participatory leadership, empowering members of the local community in leadership and decisions even in non-crisis moments (16). This includes establishing communication methods according to community norms and needs (16, 33, 41, 42). Beyond the pandemic, researchers have implemented community-based methods in public health interventions among the Latine/Hispanic community, holding public events to ascertain local needs and opinions on advance care planning (41), or delivering cancer education to be more effective and efficiently disseminated (42). In line with our findings, such interventions have identified the importance of local representatives, both in disseminating scientific information among the community and in communicating community needs and beliefs to health leaders (41, 42).

Given the degree to which members of the Latine/Hispanic community made COVID-19 decisions according to their local community and environment, we urge future pandemic researchers to employ community-based methods to both develop and disseminate knowledge/tools most effectively. We urge community-led research and relationship-development to build trust, address barriers, and decrease the inequity of health outcomes in future crises. Based on these conclusions, we identify the relevance of this study via three key actions to guide development of more equitable public health crisis preparedness plans, preventing disproportionate impact on Latine/Hispanic communities (1, 3–5).

### 5.1 Empower the values, perceptions, and priorities of the community members

Learn about the local community. Before future health crises, intentional research relationships should be established to identify community leaders, communication networks, and perceptions of health priorities, privileges, and challenges. Toward this end, we recommend methods such as community-based participatory research (CBPR), which implements intentional collaboration with community members for all steps of study development and application, increasing perceived relevance and actual efficacy of hypothetical interventions before beginning intervention design (16, 43, 44). Local public health leaders should identify key informants, community health workers, and other members of underserved communities and establish permanent lines of communication in times of calm (16). This will allow scholars and leaders to, first, gain an understanding of when, why, and how community members make health decisions. The deeper understanding of priorities, definitions of health, and avenues of information access will empower effective understanding before any intervention development begins (5.2).

### 5.2 Collaborate to maximize influence within trusted communication networks

Implement context-based intervention strategies. It is important that we accept the realities of the local community's perceptions of needs and reliable information sources. For example, among RI's Latine/Hispanic community, interpersonal agents and community members played a more significant role in decisions to participate in interventions than that of officials, scientific results, or mandates. Recognizing this, and noting the success of Promotora involvement in increasing both test-rates and psychological health during COVID-19 (16, 17, 45), we posit that including Promotoras as CBPR team members or design and implementation leaders might enable researchers to effectively address barriers of trust and information access by leading intervention design, communicating priorities, and disseminating information. We therefore urge public health policy and action leaders to prioritize collaboration with Promotoras in the development and implementation of interventions on the local health infrastructure. This might include the development of ongoing working groups or the appointment of key community members to local positions, and will allow local and health leaders to, together, develop both community-based plans for future crises and structures of bidirectional knowledge-production for non-crisis, ongoing, healthcare needs (16, 31).

### 5.3 Frame communication about interventions honestly and empower individual choice

Previous research has suggested that communication about the vaccine be clear, comprehensible, and emphasize findings on efficacy and safety, both through large-scale results and through anecdotal evidence (32–34, 46). We add that the likelihood of possible risks should be honestly communicated, and framed within the larger benefits. In addition to being developed according to knowledge about the local context (5.1) and in partnership with local leaders (5.2), policies related to interventions should protect the agency of individuals, particularly from historically underrepresented groups. We believe that full and direct communication and decision-making power, particularly within CBPR-developed and Promotora-led communication networks, will decrease fear and grant agency to community members, fostering trust through choice rather than mandate.

According to our findings, and those of community-based public health initiatives (16, 32–34), we believe that establishing ongoing bidirectional structures for both learning about local communities and providing them education, and then empowering all members to make decisions according to their own beliefs, might empower communities of trust, protecting the well-being of underserved communities during and even beyond crisis situations.

## Data Availability

Anonymized transcripts (or excerpts) generated and analyzed for this study may be provided upon request to the corresponding author.
